# Reading in the Dental Profession

**Published:** 1862-04

**Authors:** 


Reading in the Dental Profession.—There is probably no prcfession, whose members engage themselves so little in professional reading, as that of dentistry.

We make this assertion boldly, and shall proceed to substantiate it, at least inferentially, to the best of our knowledge and belief.

The profession of dentistry is originally learned almost entirely from practice. The young apprentice, in the country town or village, stands daily by the side of his employer, and following with eye and ear, obtains his first insight into the mysteries of his calling. He practices only that of which he obtains a knowledge, ocularly or orally; he sees but few books upon the subject of dentistry, for the reason that his employer learned his profession in the same manner as himself, and grew up to a belief that the world, in a dental point of view, had been at a stand-still ever since. The young man finishes his apprenticeship, and goes out into the world with this idea instilled into his mind, and the result is, that he firmly believes that dentistry is a trade like carpentry, rather than a science, to be obtained chiefly from the recorded experiences of wise practitioners.

Now it is undoubtedly true, that it is within our generation that dentistry has emerged into a science and an art, but it is for the present that we are writing, and the present generation must know that the recorded experiences upon this subject, are a library in themselves.

The difference between our profession and all others on this point is so palpable, that it never fails to strike even a layman as something strange. You enter the dwelling of a minister, or the office of a lawyer, or physician, and you find yourself surrounded with books—the scientific autobiography of the past. To be sure, it may be answered that these professions are more ancient than ours, and it is true as far as progress is concerned; but that only applies to our forefathers, and can be no excuse for those who have opportunities which they neglect.

All the true wisdom of this world is contained in books, and all the results of dental practice, down to our own day, may be found in books if properly searched for.

Who are those men in the profession who have reached the highest rank and position? They will be found to be those who have read, and those who have written. We could give many names to support this assertion, but the selection might seem invidious, and we will leave it to each one to make it for himself. The literature of dentistry may seem at first thought, to be meagre; but we can assure our readers, that on the consrary, it is exceedingly rich, when considered in connection with its youth.

There are historical works, in which the progress of the profession may be traced from the earliest period to the present time. There are biographies, where we may learn the trials, struggles, successes and defeats of men who have toiled and labored, in the same path, and the same pursuit as ourselves; there are text-books wherein the learner may obtain the first principles of his calling, and more advanced guides which contain the observations of the wisest teachers. There are, too, books which present the most complicated cases, with their treatment, and illustrate the most remarkable phases of dental disease and facial deformity. So that the earnest reader may begin at the first round of the ladder, and trace his profession to its present highest condition of success and prosperity. Lastly, there are the current periodicals,—the instruments that photograph the passing currents of dental originality, transient or permanent—and lay them before us, monthly or quarterly, or semi-annually, holding up to us a mirror wherein we may see reflected the thoughts and acts of the workers and thinkers of our profession.

But many will say, "We can not afford to own a library, and if we had one, we could not spare the time to read it." Now there are not any who can not afford to keep *au courant* with the doings of their own day; in fact, there are none who can afford to remain in ignorance of them.

As for time, suppose a dentist were to devote half an hour *per diem* to reading matters connected with his profession; in one year it would amount to thirteen working days—sufficient time to read all the periodicals published in the country. Possibly, in so reading, he might meet with much trash, but he might also meet with one article which would richly repay him for all his time and all his trouble. The application of a single new invention, improvement, or original idea might so change his whole course of practice as to inure materially to his benefit. And one can not make a practice of so reading the periodicals of the day without deriving benefits of some sort from them.

But, it will be asked, by what right do we accuse the dental profession of not reading? We reply, that the evidence of the truth of our assertion may be found recorded in the letters received at any of our dental depots, or by the publishers of the dental periodicals throughout the country. Those who read acquire mechanically the habit of writing properly, and of expressing themselves grammatically in good language, while it is a notorious fact that a large majority of the profession write ungrammatically, with false orthography, and no punctuation whatever. If it were merely to educate the profession up to its highest standard, reading would be a duty; but since it would also give them improved facilities for increasing their business, it does seem strange that they should neglect it.

The true cause for there being so little reading in the profession is that there is little industry. There are many dentists who work night and day, are always to be found at their posts, and always have an ample amount of patronage; and in nine cases out of ten they will be found to be those who are best read. But a large number are only to be found in their offices from ten to three, and then their time is too much occupied to even do their work well, and, of course, leaves them no leisure for reading. If such dentists should make it a habit to be in their offices ten or twelve hours daily, they would find that not only was their work done much better, their patronage increased, but also that they would have ample leisure for storing their minds with all the improvements and discoveries appertaining to their profession. But a dentist should not be satisfied with acquiring information alone; he should be equally ready to dispense it. If he makes some discovery, or new application, or invention it should be his pride to spread the knowledge of its usefulness throughout the profession. How easy it is to write a short description of the novelty, whatever it may be, and send it to the editor of the nearest periodical, who, if it is really useful and desirable, will be only too glad to make it public; thus the DENTAL JOURNAL becomes a medium of intercommunication in the profession, giving to the whole the benefit of the wisdom of each. It may be thought that in thus advocating the extension of reading in the profession, we are, after all, only puffing our publication; but the truth is we only desire that the dentists shall accustom themselves to reading *some* periodical, and are perfectly willing to let the selection rest with themselves, leaving the NEW YORK DENTAL JOURNAL to stand or fall on its own merits. Undoubtedly, in the case of a general dissemination of a taste for reading among the members of the dental profession, we might come in ultimately for two or three new subscribers; but the pecuniary emolument arising therefrom would not probably pay for printing this article, so we think the charge of self-interest falls to the ground by its own weight.

We have at heart the true interests of the profession, as we understand them; and we think every issue of the JOURNAL contains evidence of the justness of our intentions; and when we urge the profession to read more, it is because we believe such a course will tend to elevate them both socially and professionally, and will also largely increase their facilities for working properly, and thus increase their patronage and consequent success in life.

One more proof of our assertion we wish to add, before closing this article, and to which every editor of a dental periodical can bear witness. There is hardly a day passes over our head that we do not receive letters inquiring about some improvement, or some new discovery in the dental practice, which has, in all probability, been published and republished in all the dental journals in this country and Europe; and, to cap the climax, along comes a letter of inquiry about some subject upon which we had spent hours racking our brain in tryir g to make plain to those who choose to read our articles, and which we had taken pains to publish and place before the profession. Had these persons been in the habit of taking a dental journal, and not been content in taking it only, but spent time enough to read it, they might have spared the unfortunate editor from a repetition of what he had written, and saved themselves the shame of exposure. We say this in all kindness, not meaning to dissuade any one from writing and inquiring about what they are not familiar with, as we are always happy to give all information in our power that will tend to promote the good of the profession, or enlighten or improve any individual member; and we are willing at all times to spend a reasonable amount of our time in replying to letters, as we feel that there may be a possibility of giving some information that may result in the alleviation of pain to some human being, and because it shows that there is a disposition to inquiry upon subjects that they have not had an opportunity to become acquainted with, and a determination not to proceed in an operation in which doubt may exist as to the final result. But would it not be well for such dentists to spend a few dollars for a dental journal, besides the postage stamp that must be used in correspondence? How often have we been pained to hear dentists when asked if they take a dental journal, reply that they do not, for the reason that they are a humbug, there is nothing new in them, and in fact they know more than all the books on the subject of dentistry could teach. We have a case in point. A young man came into our editorial sanctum one day, and after conversing with him a short time, we found where he hailed from, with whom he had studied his profsssion, and what his opportunities had been for acquiring his knowledge. As he rose to leave us, we asked him if he took any dental journal, and if not, whether we should have him added to our list of subscribers. He at once informed us that he knew all that was necessary to be known, and that he could learn nothing from a dental journal! We made him a present of one, and begged him to read a few pages. We said to ourselves as he departed, "Where ignorance is bliss, 'tis folly to be wise." Happening to know his teacher, and having had an opportunity of learning the extent of his library, which consisted of Harris' Dictionary of Dental Science and Operative Dentistry, and one or two dental journals, besides the *New York Dental Journal*, to which he subscribed one year, upon condition that we would let him have it for one dollar and fifty cents, or fifty cents off for cash, we concluded he had imbibed more of the smallness of his teacher than that knowledge of the dental art that this day of enlightenment requires of all who undertake its grave responsibilities.

We could only congratulate ourselves that we were not his patient, and pity all who were so unfortunate as to fall into his hands. Thank God, for the sake of humanity, that such cases are becoming less and less common. The people read, if the dentist does not; and they are becoming so familiar with dentistry and its importance, that these ignorant machines are forced to read and understand, or starve and quit the profession, as the public will not patronize any except they be capable, faithful and honest.

Again, we have those who we know read, from the fact that they have a fine practice. They subscribe for not one but all the works that are published that can throw any light upon the profession, or that will improve the mental powers, and qualify them not only as dentists, but as gentlemen of refinement and culture, which is a qualification that no dentist can afford to be without; and, for all these publications, they appreciate the efforts of the editors and publishers, and will insist upon paying their subscriptions in advance, well knowing that it requires money to place these publications in their hands, and they are ready and willing to bear their part of the burden. They also insist upon writing and placing upon record their experience, for the benefit of those who take the trouble to peruse what they write. To such men the world owes much of its progress in our young profession, (young, comparatively speaking), and to these men all look with respect, and wonder why or how they should be so much superior to others—*the secret is, they read!*—*N. Y. Den. Jour.*

